# Drug repurposing strategies for stunting and malnutrition via malabsorption syndrome: an integrated bioinformatics approach

**DOI:** 10.1016/j.jgeb.2026.100839

**Published:** 2026-10-30

**Authors:** Lalu Muhammad Irham, Dwi Hilda Putri, Bramadi Arya, Wirawan Adikusuma, Rita Maliza

**Affiliations:** a Faculty of Pharmacy, Universitas Ahmad Dahlan, Yogyakarta, Indonesia; b Faculty of Pharmacy, Silpakorn University, Nakhon Pathom, Thailand; c Department of Biology, Faculty of Mathematics and Natural Science, Universitas Negeri Padang, West Sumatera, Indonesia; d Department of Medical Laboratory Technology, Syedza Saintika University Padang, West Sumatra, Indonesia; e Research Center for Computing, Research Organization for Electronics and Informatics, National Research and Innovation Agency, Cibinong*,* Indonesia; f Department of Biology, Faculty of Mathematics and Natural Sciences, Universitas Andalas, Padang, Indonesia

**Keywords:** Drug repurposing, Malabsorption syndrome, Stunting, Bioinformatics, Pharmacogenomics, *FOXP3*

## Abstract

Malabsorption syndrome, particularly in the context of Environmental Enteric Dysfunction (EED), has been increasingly recognized as a key underlying mechanism contributing to persistent stunting, especially in low- and middle-income countries such as Indonesia. However, its molecular basis and therapeutic potential remain inadequately explored. In this study, we applied an integrative bioinformatics approach to systematically characterize the clinical development landscape, identify key molecular targets, and explore pharmacogenomic evidence to support drug repurposing strategies for malabsorption syndrome. Data were compiled from multiple genomic, functional, and pharmacological databases, including GWAS Catalog, ClinVar, OMIM, ChEMBL, DrugBank, and the Human Protein Atlas. Drug–target–clinical trial relationships were visualized to evaluate therapeutic trends, while gene prioritization was performed based on cross-database consistency. Tissue-specific RNA and protein expression profiles were also analyzed, and pharmacogenomic associations were assessed using curated resources. The analysis revealed a diverse but still evolving therapeutic landscape, dominated by small molecules and biologics targeting immune-related pathways such as JAK3, BTK, IL2RB, and CCR9, with most candidates in early to mid-stage clinical trials. High attrition rates were observed among discontinued therapies, particularly those targeting integrin and cytokine pathways, highlighting challenges in clinical translation. Integrative prioritization identified *FOXP3, SI, SLC46A1, CUBN*, and *STAT1* as high-confidence candidate genes, with *FOXP3* demonstrating the most robust evidence across multiple databases. Tissue expression analysis supported the biological relevance of these genes, showing predominant activity in immune and gastrointestinal tissues, consistent with EED pathophysiology. Pharmacogenomic evaluation of the *FOXP3* rs3761548 variant indicated its association with variability in drug response, particularly for methotrexate and tacrolimus, including both efficacy and renal toxicity outcomes, although the evidence remains moderate and sometimes inconsistent. These findings highlight malabsorption syndrome as a promising and druggable target within the stunting pathway and emphasize the critical role of immune–epithelial interactions in its pathogenesis. The integration of multi-omics and clinical data provides a strong foundation for prioritizing therapeutic targets and supports the potential of drug repurposing as an accelerated strategy for intervention. Nevertheless, further experimental validation and population-specific studies are required to confirm these findings and facilitate their translation into clinical practice.

## 1 Introduction

Stunting remains a critical public health crisis in Indonesia, despite sustained intervention efforts. Although Stranas Stunting is centered on nutrition-specific interventions, clinical trial evidence suggests that their impact on stunting is modest, and the effectiveness of nutrition-sensitive interventions in promoting child growth remains insufficiently demonstrated.^1^ More broadly, although child survival has improved, a substantial number of young children worldwide continue to experience developmental challenges, including stunting, wasting, and micronutrient deficiencies.^2^ The immediate and long-term consequences of stunting are well established, with the most significant impact being on children's developmental outcomes.^3^ Chronic insufficient energy and nutrient intake, together with recurrent infections, are established contributors to childhood undernutrition; however, additional factors also play an important role and should be considered when designing stunting-reduction programs. As a result, a multisectoral approach to addressing stunting has increasingly gained attention in recent years.^4^ Malabsorption syndrome, characterized by chronic nutrient deficiencies due to impaired intestinal absorption, serves as a major pathophysiological driver of linear growth failure.^5^ This condition creates persistent deficits in essential micronutrients—particularly zinc for growth signalling, protein for tissue synthesis, vitamin A for epithelial maintenance, and iron for metabolic function—disrupting the insulin-like growth factor 1 (IGF-1) pathway critical for height development. Environmental Enteric Dysfunction (EED), marked by subclinical villous atrophy and intestinal inflammation, further exacerbates this malabsorption-stunting axis, rendering conventional WASH interventions insufficient without targeted nutritional therapies.^6^ In this study, malabsorption syndrome was used as a computational phenotype for identifying molecular and pharmacological evidence relevant to impaired intestinal nutrient absorption. Malabsorption syndrome, EED, and childhood stunting are distinct clinical and biological entities and should not be considered interchangeable. EED may contribute to impaired intestinal function and nutrient utilization, which may in turn contribute to growth faltering, but the relationship is multifactorial and cannot be established as causal using the present database-based analysis.

The complexity of malabsorption-driven stunting demands innovative therapeutic strategies beyond traditional supplementation.^7^ Drug repurposing offers a rapid, cost-effective alternative by leveraging existing pharmacologically characterized compounds with established safety profiles.^8^ For example, drug repurposing has been applied across various diseases, as demonstrated in the work by Irham et al and colleagues, who explored its potential in different therapeutic contexts.^9^, ^10^, ^11^, ^12^, ^13^ In addition, bioinformatics-driven drug repurposing can systematically identify candidate drugs by integrating genomic variants associated with multiple diseases, along with pharmacogenomic data and clinical trial evidence, to prioritize compounds relevant to malabsorption pathways. Recent advances have further expanded the computational landscape of drug repurposing by integrating machine learning, deep learning, molecular docking, virtual screening, network-based approaches, and disease-associated molecular signatures. These approaches have been successfully applied across diverse disease contexts, including COVID-19, virus-associated cancers.^14^, ^15^Therefore, current study uses a bioinformatics-driven approach to identify candidate drugs through integrative analysis of genomic associations, pharmacogenomic data, and clinical trial outcomes specific to malabsorption pathways.

This study employs Open Targets Platform data (EFO_0009554, accessed February 2026) integrated with ChEMBL, DrugBank, PharmGKB, and Human Protein Atlas resources to systematically map repurposing opportunities. We sincerely thank the Open Targets team for providing an open-access platform that facilitates the integration and interpretation of target–disease associations, thereby supporting the systematic prioritization of candidate genes and therapeutic hypotheses. By combining genomic evidence, druggability assessments, and clinical trial outcomes, we identify pharmacologically actionable targets within the malabsorption-stunting pathway. The findings provide a hypothesis-generating framework for prioritizing molecular pathways and pharmacological candidates for subsequent investigation in EED-associated malnutrition and stunting.

Current pharmacotherapeutic development for malabsorption syndrome reveals a diverse clinical landscape dominated by small molecules and biologics targeting immune signalling (*JAK3, BTK, IL2RB, CCR9*) and epithelial barrier function. This analysis reveals untapped opportunities within approved drugs for repurposing applications in stunting prevention. Integrative multi-omics prioritization identified *FOXP3, SI, SLC46A1, CUBN*, and *STAT1* as high-confidence genetic targets supported across GWAS, ClinVar, OMIM, and functional genomic databases. *FOXP3* demonstrates particularly robust evidence, with pharmacogenomic associations linking the rs3761548 variant to variable responses in methotrexate and tacrolimus therapies. Tissue expression profiling confirms predominant activity in immune tissues (blood, spleen) and gastrointestinal epithelium, validating biological relevance to malabsorption pathophysiology. These findings establish a prioritized gene-drug-target framework for systematic repurposing.

## 2 Methods

### 2.1 Data acquisition and sources

This study employed an integrative bioinformatics approach utilizing publicly available genomic, pharmacogenomic, and clinical trial databases accessed on February 10, 2026. Primary data for malabsorption syndrome (EFO_0009554) were retrieved from Open Targets Genetics Platform (https://platform.opentargets.org/disease/EFO_0009554), providing comprehensive genetic associations, known drug interactions, and druggability scores. Clinical trial and mechanism-of-action data were curated from ChEMBL database (version 35) and validated through ClinicalTrials.gov (https://clinicaltrials.gov/), capturing investigational and approved drugs associated with malabsorption syndrome across all development phases and trial statuses. Supplementary pharmacogenomic evidence was extracted from PharmGKB (https://www.pharmgkb.org/), the ClinPGx database (https://www.clinpgx.org/), and DrugBank (version 6.0). Functional genomic annotations were obtained from GWAS Catalog, ClinVar, OMIM, UniProt, GeneBurden, Genebass, and ClinGen. Tissue-specific expression profiles were derived from Expression Atlas (RNA-level) and Human Protein Atlas (protein-level, immunohistochemistry data). To support biological interpretation, tissue-specific expression profiles were retrieved from Expression Atlas at the RNA level and from the Human Protein Atlas at the protein level using immunohistochemistry data. Together, these complementary resources enabled a comprehensive multi-layered analysis of genetic susceptibility, pharmacological relevance, and tissue expression, thereby supporting robust target prioritization for malabsorption syndrome.

### 2.2 Clinical trial data analysis and sankey visualization

Drug candidates associated with malabsorption syndrome were systematically identified through ChEMBL and clinicaltrials.gov queries filtering for clinical trial records (Phases 1–4) and regulatory approvals. Data extraction included drug names, molecular types (small molecules, proteins, antibodies), target genes/proteins (HGNC symbols), trial phases, status (completed, recruiting, terminated, withdrawn), and clinical trial identifiers (NCT numbers). Sankey diagrams (Fig. 1, Fig. 2) were generated using RAWGraphs (https://app.rawgraphs.io/) to visualize the multi-step relationships among drugs, molecular types, targets, trial phases, status, and trial identifiers. This visualization was chosen because it allows complex many-to-many associations to be displayed in a clear and intuitive flow format, where the width of each connection represents the relative strength or frequency of the link. In this study, the Sankey diagrams help summarize how each drug is connected to a specific molecular type, which in turn is linked to one or more targets, then mapped to clinical trial phases, trial status, and finally to individual trial identifiers. By arranging the data in this sequential manner, the diagrams make it easier to trace the pathway from a candidate compound to its clinical development context, thereby highlighting both direct and indirect relationships that may not be obvious in tabular form.

**Fig. 1:**
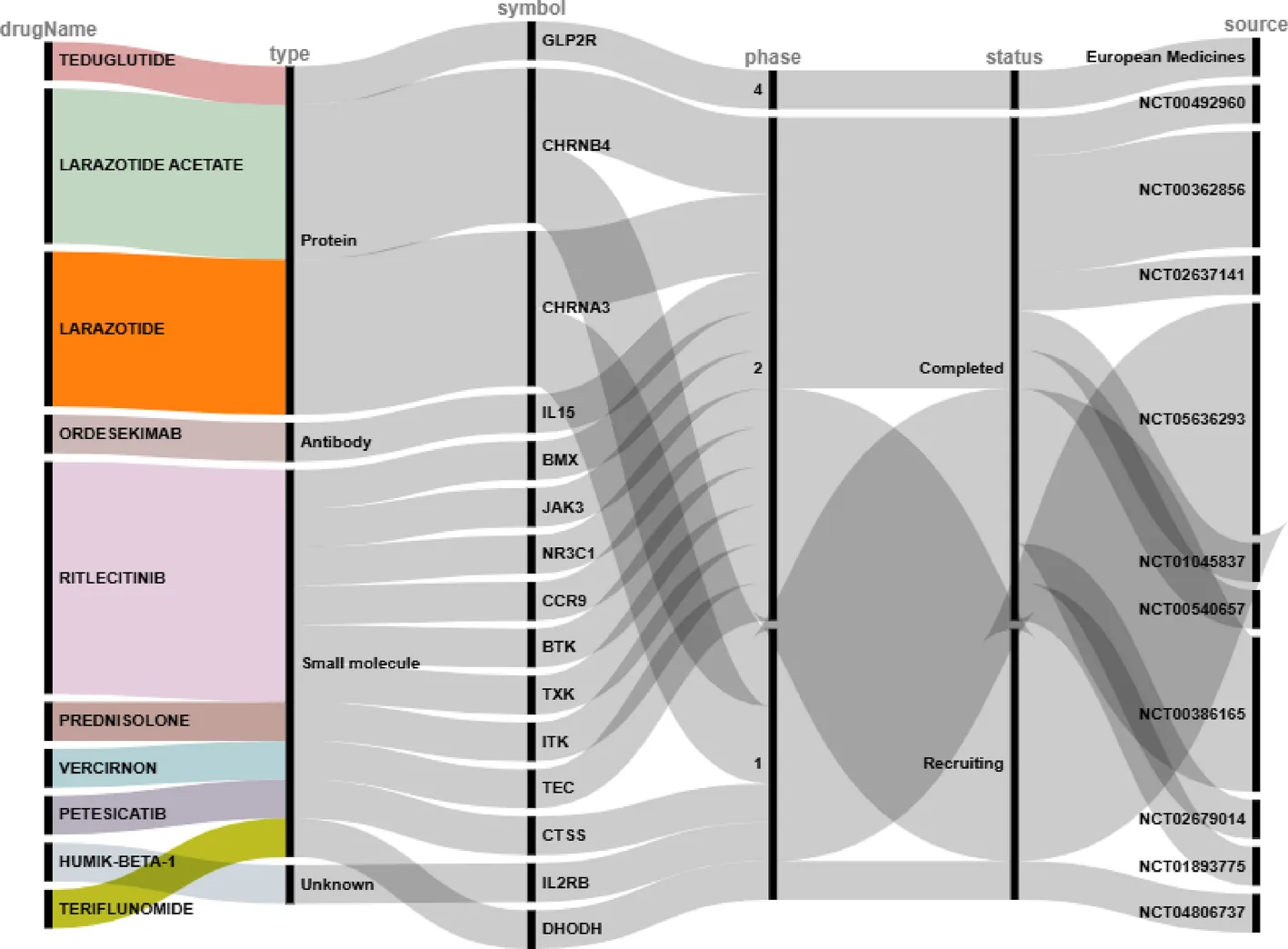
Clinical precedence for investigational or approved drugs indicated completed and recruiting for malabsorption syndrome and curated mechanism of action. This Sankey diagram illustrates the relationships between drugs, their molecular types, target genes/proteins, clinical development phases, trial status, and data sources for therapies associated with malabsorption syndrome. From left to right, the diagram displays: (i) drug names, (ii) drug type classifications (e.g., protein, antibody, small molecule), (iii) molecular targets (gene symbols), (iv) clinical trial phases, (v) trial status (e.g., completed, recruiting), and (vi) corresponding clinical trial identifiers or regulatory sources. The width of the connecting flows represents the relative number or strength of associations between each category. This visualization integrates curated mechanism-of-action data and clinical trial information derived from the ChEMBL database, providing a comprehensive overview of therapeutic development pathways.

**Fig. 2:**
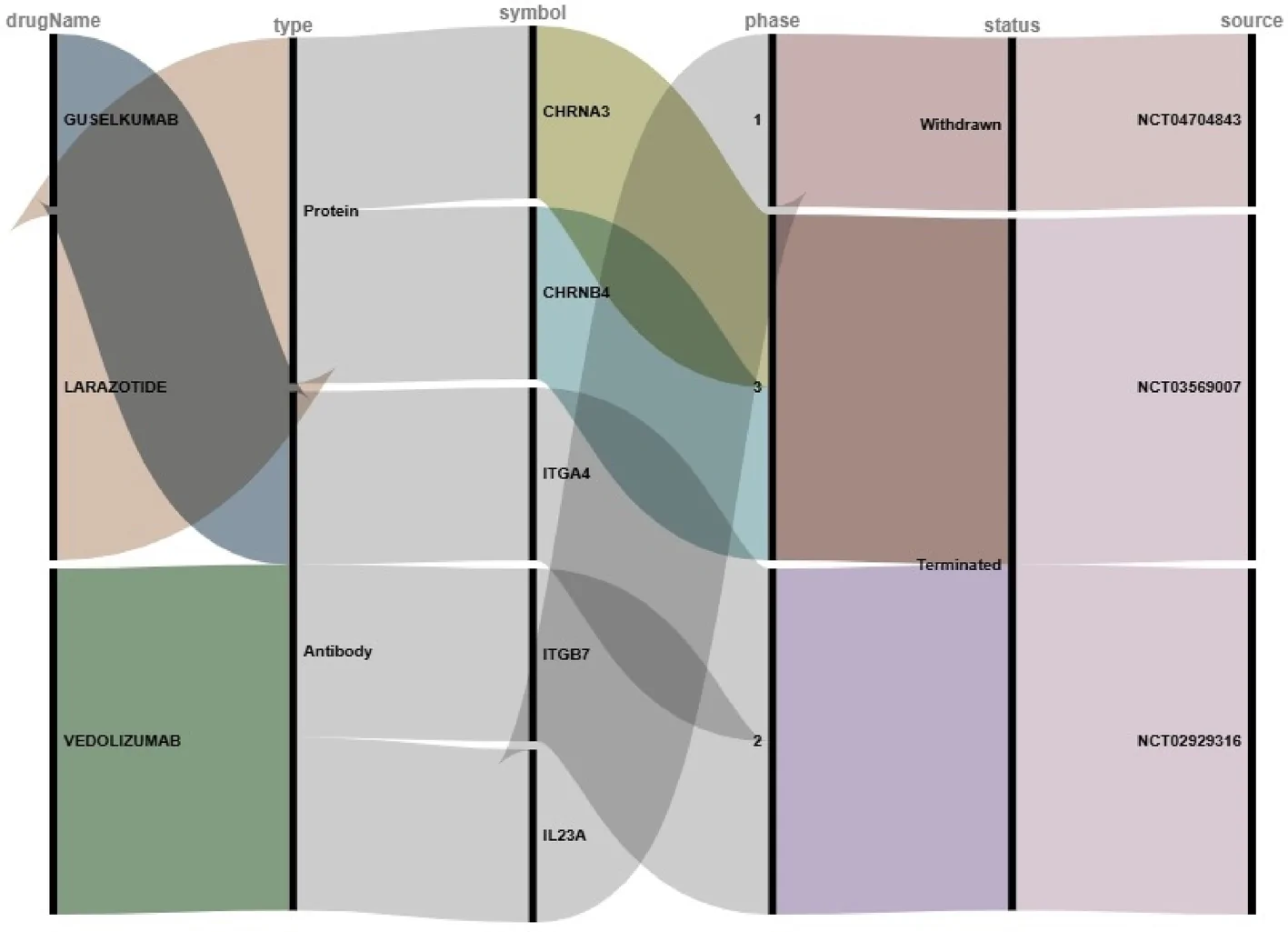
Clinical precedence for investigational or approved drugs indicated in terminated or withdrawn clinical trials for malabsorption syndrome and curated mechanism of action. This Sankey diagram illustrates the relationships between drug candidates, their molecular types, target genes/proteins, clinical trial phases, trial status, and corresponding clinical trial identifiers for therapies associated with malabsorption syndrome that were either terminated or withdrawn. From left to right, the diagram displays: (i) drug names, (ii) drug type classifications (e.g., protein, antibody), (iii) molecular targets (gene symbols), (iv) clinical development phases, (v) trial status (terminated or withdrawn), and (vi) clinical trial identifiers. The width of the connecting flows represents the relative number or strength of associations between categories.

These figures are particularly useful for identifying patterns such as drugs associated with multiple targets, targets that appear across different trial phases, and compounds that have progressed to advanced clinical statuses. In addition, the interactive and customizable features of https://app.rawgraphs.io/ make it possible to refine node labels, colors, spacing, and link thickness for improved readability and presentation quality. Collectively, the Sankey diagrams provide a compact visual summary of the translational landscape, supporting interpretation of drug repurposing opportunities and clinical evidence integration.

### 2.3 Integrative multi-database gene prioritization

Candidate gene prioritization was performed using an integrative evidence framework incorporating genomic, functional, disease-association, expression, and pharmacological resources. Candidate genes were evaluated across five evidence domains: (i) genetic association, including GWAS Catalog, GeneBurden, and ClinVar; (ii) somatic alterations, including GEL PanelApp, Gene2Phenotype, UniProt literature and curated variants, and Orphanet; (iii) functional genomics, including ClinGen, Cancer Gene Census, IntOGen, somatic ClinVar annotations, and cancer biomarkers; (iv) associated models, including ChEMBL, CRISPR screens, Project Score, and Reactome; and (v) literature and other evidence, including Europe PMC, Expression Atlas, and MPC Fig. 3. Evidence from individual databases was standardized to facilitate comparison across heterogeneous data sources. No subjective database-specific weighting was applied; instead, prioritization was based on the convergence and consistency of evidence across independent resources. Genes supported by evidence from at least five databases were considered high-confidence candidates. This approach was designed to prioritize biologically supported candidate genes based on cross-database evidence convergence rather than to estimate a quantitative probability of therapeutic efficacy.

For each candidate gene, evidence retrieved from the predefined databases was transformed into a standardized score to facilitate comparison across heterogeneous evidence types. No differential weighting was assigned to individual databases; each database contributed equally to the integrated evidence framework. Missing values were treated as unavailable evidence and were not interpreted as evidence against a gene. The Open Targets association score (>0.8) was applied as an initial inclusion criterion and was not used as an additional database weight. Genes supported by evidence from ≥5 predefined resources were classified as high-confidence candidates, whereas consistency across ≥3 independent resources was used as a quality-control criterion. Standardized evidence intensity was visualized using a purple-gradient heat map, where darker shades represented stronger or more consistent evidence across the evaluated resources. The complete list of candidate genes identified before prioritization is provided in Supplementary Table S1, together with the corresponding database-specific evidence and integrated prioritization scores. The resulting prioritization identified *FOXP3,* *SI, SLC46A1,* *CUBN,* and *STAT1* as high-confidence candidate genes for subsequent tissue-expression and pharmacogenomic analyses (Fig. 3).

**Fig. 3:**
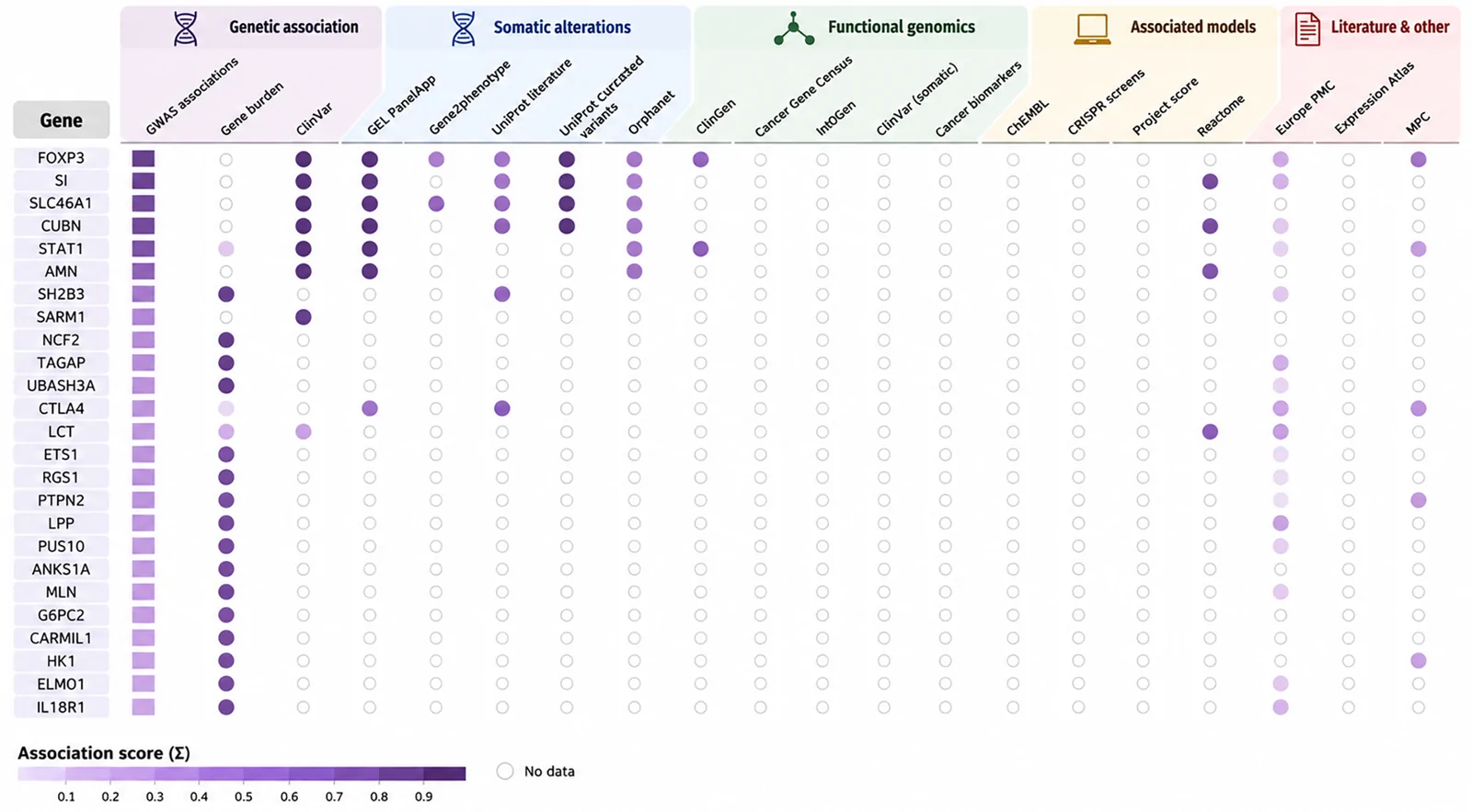
Integrative multi-source evidence landscape for candidate gene prioritization. Candidate genes were evaluated across five evidence domains: (i) genetic association, including GWAS Catalog, GeneBurden, and ClinVar; (ii) somatic alterations, including GEL PanelApp, Gene2Phenotype, UniProt literature and curated variants, and Orphanet; (iii) functional genomics, including ClinGen, Cancer Gene Census, IntOGen, somatic ClinVar annotations, and cancer biomarkers; (iv) associated models, including ChEMBL, CRISPR screens, Project Score, and Reactome; and (v) literature and other evidence, including Europe PMC, Expression Atlas, and MPC. Each row represents a candidate gene, and each column represents an evidence source. Adapted from Open Targets Platform, with modifications to the graphical presentation and color scheme.

### 2.4 Tissue expression profiling

Tissue-specific transcriptomic and proteomic analysis. Tissue-level RNA expression data were obtained from Expression Atlas, while protein expression information was retrieved from the Human Protein Atlas (HPA). The Open Targets Platform supported the broader study by facilitating the integration and interpretation of target–disease associations, complementing the transcriptomic and proteomic evidence used to prioritize candidate genes. As shown in Fig. 4 the left panel presents RNA expression across tissue categories using blue gradient bars, with the colour intensity representing relative expression levels from high to low. The right panel displays protein expression using teal bars, with the bar length indicating the relative level of protein abundance from low to high. Tissues are arranged vertically to facilitate comparison between transcriptomic and proteomic profiles. “N/A” indicates that data were unavailable for the corresponding tissue. This comparative visualization provides an overview of tissue-specific expression patterns and enables assessment of the concordance and potential discrepancies between RNA and protein abundance across tissues.

**Fig. 4:**
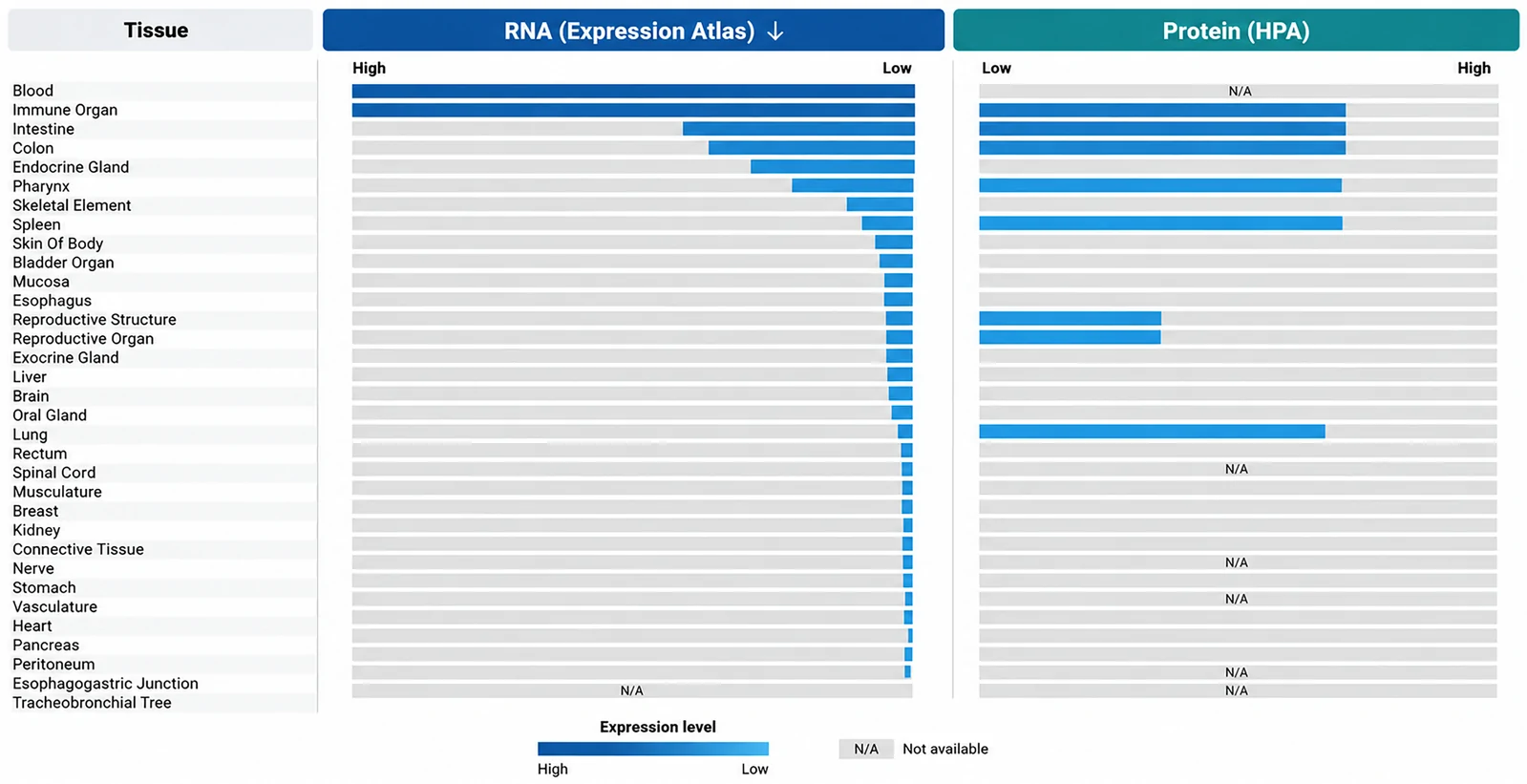
Tissue-specific RNA and protein expression profiles across human organs based on Expression Atlas and Human Protein Atlas data. This figure illustrates the tissue-specific expression pattern of the selected gene across a wide range of human tissues. The left panel represents RNA expression levels derived from the Expression Atlas, while the right panel shows protein expression levels based on data from the Human Protein Atlas (HPA). Each row corresponds to a specific tissue type, and horizontal bars indicate relative expression levels, ranging from low to high. Blue bars represent detected expression, with increasing intensity corresponding to higher expression levels, while grey bars indicate low or undetected expression. “N/A" denotes unavailable data for the respective tissue and dataset. RNA expression is broadly quantified across tissues, whereas protein expression reflects immunohistochemistry-based detection and may show discrepancies due to post-transcriptional regulation, protein stability, or data availability. This visualization enables comparison between transcriptomic and proteomic expression landscapes across tissues. Adapted from Open Targets Platform, with modifications to the graphical presentation and color scheme.

### 2.5 Pharmacogenomic analysis

FOXP3 rs3761548 variant analysis (Table 1) extracted all documented drug response associations from the ClinPGx database, filtering for efficacy/toxicity phenotypes with confidence ≥Level 3. Data fields included SNP identifier, associated drugs (methotrexate, tacrolimus), response phenotype (increased/decreased efficacy, toxicity risk), category, and evidence level. Response heterogeneity was quantified by conflicting effect directions across studies. Indirect pharmacogenomic effects were noted where *FOXP3* represents pathway-level rather than direct drug target interactions.

**Table 1:** Genetic variants in ***FOXP3*** that have been associated with drug response (Gene may not be direct target of the drug).

SNP	Drug(s)	Drug Response Phenotype	Drug Response Category	Confidence Level
rs3761548	methotrexate	increased response	efficacy	Level 3
rs3761548	tacrolimus	increased risk of acute renal toxicity	toxicity	Level 3
rs3761548	methotrexate	increased response	efficacy	Level 3
rs3761548	methotrexate	decreased response	efficacy	Level 3
rs3761548	tacrolimus	increased risk of acute renal toxicity	toxicity	Level 3
rs3761548	methotrexate	decreased response to methotrexate	efficacy	Level 3
rs3761548	tacrolimus	decreased risk of acute renal toxicity	toxicity	Level 3

### 2.6 Data integration and bioinformatics pipeline

All analyses were constructed using Open Targets association scores (>0.8 threshold). Druggability assessment combined Open Targets tractability (A/B categories), ChEMBL target confidence (high/medium), and DrugBank approval status.

### 2.7 Quality control and validation

Data quality filtering excluded associations with confidence levels below Level 3, phase 0 or preclinical evidence, and statistically nonsignificant associations (*p* > 0.05). Candidate genes were prioritized based on concordant evidence across databases, integrating genetic associations, pharmacogenomic evidence, drug–target interactions, clinical trial information, and tissue-specific expression data. This approach supported the systematic identification of biologically relevant therapeutic targets and potential drug repurposing candidates for malabsorption syndrome. Analytical code, filtered datasets, and visualization scripts were retained to support reproducibility. This study analyzed publicly accessible genomic, pharmacogenomic, and clinical trial databases without recruiting human participants or collecting new personal data. Therefore, ethical approval and informed consent were not required, subject to applicable institutional policies and database access requirements.

## 3 Results

### 3.1 Clinical development landscape and target mapping of investigational and approved drugs for malabsorption syndrome

The integrative analysis of drug–target–clinical trial relationships revealed a diverse therapeutic landscape for malabsorption syndrome, encompassing multiple drug classes and molecular targets. The Sankey diagram demonstrates that small molecules constitute the majority of investigated compounds, followed by biologics such as proteins and antibodies. Several drugs, including larazotide acetate and ritelcitinib, are prominently represented and linked to multiple molecular targets, reflecting their broader mechanistic roles. Target mapping highlights key genes such as *JAK3, BTK, IL2RB, and CCR9*, suggesting that immune signalling pathways play a central role in therapeutic strategies for this condition.

In terms of clinical development, most drug candidates are concentrated in early to mid-stage trials (Phase 1 and Phase 2), with fewer compounds reaching Phase 4, indicating that the field is still evolving with limited late-stage or post-marketing evidence. Trial status distribution shows a substantial proportion of studies in the recruiting phase, alongside a smaller number of completed trials, highlighting ongoing research efforts and active clinical investigation. The presence of multiple clinical trial identifiers linked to specific drug–target pairs underscores the heterogeneity of study designs and therapeutic approaches. This analysis reveals that current drug development for malabsorption syndrome is target-diverse but still in relatively early clinical stages, with a strong emphasis on immune-related mechanisms. These findings provide insight into potential therapeutic targets and highlight opportunities for further drug development and clinical validation.

### 3.2 Clinical development landscape of terminated and withdrawn drug candidates for malabsorption syndrome

The analysis of terminated and withdrawn drug candidates for malabsorption syndrome highlights key patterns in clinical attrition and target selection. The Sankey diagram demonstrates that both protein-based therapies and monoclonal antibodies have experienced discontinuation, indicating that attrition is not limited to a single drug modality. Several prominent candidates, including larazotide, vedolizumab, and guselkumab, were linked to multiple molecular targets such as CHRNA3, CHRNB4, ITGA4, ITGB7, and IL23A, reflecting a focus on epithelial barrier function and immune-mediated pathways. These targets are consistent with mechanisms involving intestinal permeability and immune regulation.

Most discontinued candidates were concentrated in early- to mid-stage clinical trials (Phase 1–3), suggesting challenges in demonstrating efficacy or safety prior to late-stage development. The presence of both terminated and withdrawn statuses indicates a range of underlying causes, including strategic, safety, or efficacy-related decisions. Notably, integrin-related targets (e.g., *ITGA4* and *ITGB7*) and cytokine-associated pathways (e.g., *IL23A*) were recurrent among discontinued programs, which may reflect the complexity of modulating immune responses in malabsorption-related conditions. We highlighted, these findings suggest that while immune and barrier-related targets remain central to therapeutic strategies, clinical translation remains challenging, with significant attrition occurring before late-phase validation. This underscores the need for improved target validation, patient stratification, and trial design to enhance success rates in future drug development efforts.

The integrative analysis identified several genes with consistent support across multiple data sources, highlighting their potential relevance in the studied condition. Genes such as *FOXP3,* *SI, SLC46A1,* *CUBN*, and *STAT1* demonstrated strong and recurrent signals across genomic association datasets (e.g., GWAS Catalog, GeneBurden, and Genebass), as well as functional annotations (UniProt variants and OMIM), indicating robust genetic and biological relevance. Notably, *FOXP3* exhibited the most comprehensive evidence profile, with high-intensity associations spanning multiple genomic and functional databases, suggesting a central role in disease mechanisms. The complete candidate-gene dataset is provided in Supplementary Table S1. Following application of the predefined prioritization criteria, *FOXP3, SI, SLC46A1, CUBN,* and *STAT1* were identified as high-confidence candidate genes (Fig. 3). In contrast, several genes such as *MLN, G6PC2*, and *HK1* displayed more limited or database-specific evidence, indicating either context-dependent roles or gaps in current knowledge.

Pharmacological database integration (e.g., ChEMBL, DrugBank, PharmGKB) revealed that only a subset of prioritized genes has known drug interactions, underscoring potential opportunities for drug repurposing or novel therapeutic targeting. Additionally, expression-based resources such as the Human Protein Atlas supported tissue-specific relevance for several high-priority genes. Overall, the cross-database consistency observed for top-ranked genes reinforces their prioritization, while discrepancies across data types highlight areas requiring further experimental validation. This integrative approach provides a robust framework for identifying biologically and clinically meaningful targets.

### 3.3 FOXP3 Tissue-Specific RNA and protein expression profiles across human organs

Analysis of tissue-specific expression profiles revealed a distinct pattern of RNA and protein distribution across human organs. RNA expression data demonstrated the highest expression levels in blood and immune-related tissues, followed by moderate expression in the intestine and colon, suggesting a potential role in immune and gastrointestinal biological processes. In contrast, RNA expression was generally low or negligible across most other tissues, including the brain, liver, kidney, and musculoskeletal systems, indicating a degree of tissue specificity. Protein expression data from the Human Protein Atlas partially supported these findings, with detectable protein levels observed in selected tissues such as immune organs, spleen, and lung, although the distribution appeared more restricted compared to RNA expression. Several tissues showed “N/A” values, reflecting limited protein-level evidence or lack of available data Fig. 4. Notably, discrepancies between RNA and protein expression patterns were observed, which may reflect post-transcriptional regulation, differences in detection sensitivity, or biological variation in protein stability. Integrated transcriptomic and proteomic data indicate that this gene is primarily expressed in immune and epithelial tissues, pointing to a role in immune modulation and tissue-specific physiology. Consequently, these results offer valuable insight into the gene’s biological significance and its viability as a therapeutic target.

### 3.4 Pharmacogenomic associations of *FOXP3* genetic variant (rs3761548) with drug response phenotypes

The pharmacogenomic analysis identified multiple associations between the *FOXP3* SNP rs3761548 and variability in drug response, particularly for methotrexate and tacrolimus. Across the compiled evidence, rs3761548 was linked to both efficacy and toxicity outcomes, although all associations were supported by moderate-level evidence (Level 3). For methotrexate, rs3761548 demonstrated inconsistent effects on treatment efficacy, with reports indicating both increased and decreased therapeutic response. This variability suggests a complex role of *FOXP3* in modulating immune-related treatment outcomes, potentially influenced by patient-specific or disease-specific contexts.

In the case of tacrolimus, the same variant was associated with altered risk of acute renal toxicity, with studies reporting both increased and decreased risk. These conflicting findings highlight potential heterogeneity in study populations or differences in clinical settings. Overall, the evidence suggests that rs3761548 in *FOXP3* may contribute to interindividual variability in drug response, particularly for immunomodulatory therapies. However, the inconsistency of reported effects and the moderate confidence level underscore the need for further validation in larger, well-controlled studies before clinical implementation.

This table summarizes pharmacogenomic evidence linking the single nucleotide polymorphism (SNP) rs3761548 in the *FOXP3* gene with variability in drug response. The table includes the associated drug(s), observed drug response phenotypes, categorized response types (efficacy or toxicity), and the level of supporting evidence. Drug response phenotypes indicate whether the variant is associated with increased or decreased therapeutic efficacy or altered toxicity risk. The “confidence level” reflects the strength of evidence as curated in the ClinPGx database, where Level 3 represents moderate evidence derived from limited or inconsistent studies. It is important to note that *FOXP3* may not be a direct pharmacological target of the listed drugs, and associations may reflect indirect or pathway-level effects. Data were obtained from the ClinPGx database (https://www.clinpgx.org/).

## 4 Discussion

The present study provides a comprehensive and integrative perspective on the therapeutic landscape of malabsorption syndrome, positioning it as a critical mechanistic axis underlying persistent stunting, particularly in low- and middle-income settings such as Indonesia. By combining clinical trial mapping, multi-database gene prioritization, and pharmacogenomic analysis, this work extends beyond conventional nutritional and environmental frameworks and emphasizes the importance of molecular and immunological determinants of nutrient absorption.

The present study provides a novel systems-level perspective on the persistent burden of stunting in Indonesia by highlighting malabsorption syndrome—particularly in the context of Environmental Enteric Dysfunction (EED)—as a critical and potentially druggable therapeutic target. Through a multi-database bioinformatics approach, our findings consistently identified *FOXP3, SI, SLC46A1, CUBN*, and *STAT1* as high-confidence candidate genes, suggesting that disruptions in immune regulation, intestinal transport, and inflammatory signalling play central roles in impaired nutrient absorption. This bioinformatics analysis establishes malabsorption syndrome as a critical therapeutic target for Indonesia's persistent stunting crisis, identifying *FOXP3, SI, SLC46A1,CUBN*, and *STAT1* as high-confidence genes for further investigation. The prioritized genes demonstrate robust genetic associations spanning GWAS, ClinVar, and OMIM, with *FOXP3* exhibiting the strongest evidence profile across 12 resources. Tissue expression validation confirms biological relevance, particularly FOXP3's predominant immune/gastrointestinal localization aligning with EED pathophysiology. These findings validate the malabsorption-stunting axis as druggable, extending beyond WASH limitations by targeting molecular nutrient absorption deficits.

The clinical trial landscape reveals immune-modulatory pathways (JAK3, BTK, CCR9) as dominant therapeutic strategies, yet high attrition rates among integrin inhibitors (ITGA4/ITGB7) and anti-cytokine therapies underscore translational challenges.^16^, ^17^, ^18^ Among barrier-directed candidates, larazotide acetate is particularly notable because it modulates intestinal epithelial tight junctions and has been investigated for reducing intestinal permeability and immune activation in celiac disease models and clinical studies. This pattern reflects the complexity of intestinal barrier–immune crosstalk in malabsorption, where single-target approaches may be insufficient to address multifactorial nutrient losses. Importantly, the identified compounds should not be interpreted as established treatments for stunting. Their potential relevance to stunting is indirect and hypothesis-generating, based on their reported relationships with pathways implicated in intestinal inflammation, epithelial barrier function, and nutrient absorption. Whether modulation of these pathways can improve nutrient status or ultimately influence linear growth in children with EED-associated stunting remains unknown and requires dedicated preclinical and clinical investigation. Drug repurposing may nevertheless provide a useful framework for prioritizing existing pharmacologically characterized compounds for further investigation.^19^

*FOXP3* emerged as the highest-confidence candidate in our computational prioritization; however, this finding should not be interpreted as evidence that FOXP3 is a validated therapeutic target. In renal transplant patients, FOXP3 rs3761548 was associated with tacrolimus-induced acute nephrotoxicity, with AA and AC genotypes conferring a markedly higher risk than the CC genotype, indicating that this variant can influence clinically relevant immune-drug responses in a context-dependent manner.^20^ Although these findings were obtained in a disease setting different from stunting, they support the broader concept that FOXP3 variation may modulate immune regulation and treatment response across inflammatory conditions.^21^ In our study, through bioinformatic approach we noted that the rs3761548-linked pharmacogenomic signal suggests potential relevance to malabsorption-driven stunting, where chronic inflammation and dysregulated regulatory T-cell activity may contribute to epithelial injury and impaired nutrient absorption.^22^ However, this variant has not yet been validated in the Indonesian stunting population, and our interpretation should therefore be considered hypothesis-generating rather than definitive. The tissue-specific expression pattern, with higher expression in blood than intestine, further supports a systemic immune-regulatory role and suggests that FOXP3- high-confidence candidate gene strategies would require careful translational evaluation before clinical application.

The pharmacogenomic associations involving *FOXP3* rs3761548 should be interpreted cautiously because the available evidence was classified as Level 3 and originated from clinical contexts unrelated to EED or childhood stunting. Moreover, the reported associations showed inconsistent directions across studies and involved both efficacy and toxicity phenotypes. *FOXP3* may also represent a pathway-level biomarker rather than a direct pharmacological target of methotrexate or tacrolimus. Consequently, these findings provide biological hypotheses regarding interindividual variation in immune-related drug responses but do not provide sufficient evidence to support *FOXP3*-guided therapy or pharmacogenomic treatment selection in children with stunting.

Importantly, the analysis of terminated and withdrawn drug candidates provides critical insight into translational challenges. The discontinuation of agents such as larazotide, vedolizumab, and guselkumab—despite targeting biologically relevant pathways including epithelial barrier function (e.g., CHRNA3/CHRNB4) and immune regulation (e.g., IL23A, ITGA4/ITGB7)—suggests that targeting single mechanisms may be insufficient in addressing the multifactorial nature of malabsorption. The recurrence of integrin and cytokine-related targets among failed programs highlights the complexity of immune–epithelial interactions and suggests that therapeutic success may require combinatorial or systems-level approaches rather than single-target interventions. These findings align with previous reports indicating that intestinal permeability, inflammation, and microbiome alterations act synergistically in conditions such as Environmental Enteric Dysfunction (EED).^23^, ^24^, ^25^

At the genomic level, our integrative prioritization identified *FOXP3,* *SI, SLC46A1,CUBN*, and *STAT1* as the most consistently supported candidate genes across diverse data sources, including GWAS, ClinVar, OMIM, and functional annotation databases. Among these, FOXP3 emerged as the most robust candidate, supported by convergent evidence across genomic, functional, and pharmacological datasets. Biologically, FOXP3 plays a central role in regulatory T-cell (Treg) function and immune tolerance, which is highly relevant in the context of chronic intestinal inflammation and barrier dysfunction. FOXP3 was prioritized as the highest-confidence candidate based on the convergence of genomic, functional, expression, and pharmacological evidence across multiple databases. Its established role in regulatory T-cell function provides biological plausibility in the context of chronic intestinal immune dysregulation. However, this prioritization represents a hypothesis-generating finding and does not establish *FOXP3* as a validated therapeutic target for EED or stunting. Similarly, *STAT1* is a key mediator of cytokine signalling and immune activation, while *SLC46A1,* and CUBN are involved in nutrient transport and absorption processes. The convergence of immune-regulatory and nutrient-transport pathways supports a model in which malabsorption arises from the interplay between inflammation-driven epithelial damage and impaired nutrient uptake.^26^, ^27^, ^28^, ^29^

Tissue-specific expression analysis further strengthens the biological plausibility of these targets. The predominant RNA expression of prioritized genes in blood and immune-related tissues, with moderate expression in intestinal compartments, suggests a dual systemic and local role in disease pathophysiology. The partial concordance between RNA and protein expression profiles reflects known complexities in post-transcriptional regulation and protein stability, yet consistently points toward immune and epithelial tissues as key sites of action. This spatial expression pattern is highly consistent with the pathophysiological features of EED, where chronic immune activation and intestinal barrier disruption coexist.^30^, ^31^, ^32^ Drug repurposing nowadays become more familiar concept since many databases can be integrated for various disease.^33^, ^34^, ^35^, ^36^, ^37^, ^38^ Computational drug repurposing has increasingly evolved from single-database approaches toward integrated frameworks incorporating network-based analysis, machine learning, deep learning, molecular docking, virtual screening, and disease-signature reversal. Previous studies have demonstrated the utility of these approaches across, prostate, colorectal and endometrial cancers.^10^, ^11^, ^39^, ^40^ These studies support the broader methodological rationale for integrating heterogeneous genomic, pharmacological, and clinical evidence, as performed in the present study. However, unlike approaches primarily focused on molecular screening or predictive modelling, our framework emphasizes cross-database evidence convergence and target prioritization for malabsorption syndrome.

From a therapeutic perspective, these findings collectively support the concept of drug repurposing as a pragmatic strategy to accelerate intervention development. Given the high attrition rates observed in novel drug development, repurposing existing agents with known safety profiles—particularly those targeting immune pathways—may offer a more efficient route toward clinical translation. However, the limited druggability of certain targets, such as nutrient transporters (e.g., CUBN), highlights the need for innovative approaches, including indirect pathway modulation or combination therapies.

In our study, we used the Open Targets Platform to systematically prioritize candidate genes by integrating genetic, functional, disease-associated, and pharmacological evidence, thereby generating evidence-informed therapeutic hypotheses^41^. However, its prioritization depends on the availability and quality of underlying data and does not establish causality. Reliance on in silico analyses and the absence of experimental validation or an independent disease-specific cohort limit mechanistic interpretation and external validation. Moreover, potential population bias in genomic databases may restrict generalizability to Indonesian populations, while discrepancies between transcriptomic and proteomic data and incomplete pharmacological annotations highlight existing knowledge gaps. Future studies incorporating Indonesian population-specific clinical and genomic data, independent cohorts, and experimental validation are needed to confirm the prioritized genes and pharmacogenomic associations in children with EED-associated stunting.

## 5 Conclusion

In conclusion, this study provides a robust, multi-dimensional framework for understanding malabsorption syndrome as a key contributor to stunting, integrating clinical, genomic, and pharmacological evidence. The identification of high-confidence targets such as *FOXP3, SI, SLC46A1, CUBN*, and *STAT1*, together with insights into clinical development challenges and pharmacogenomic variability, offers a strong foundation for future translational research. Moving forward, experimental validation, population-specific studies, and well-designed clinical trials will be essential to translate these findings into effective therapeutic strategies aimed at addressing the complex and multifactorial burden of stunting.

## CRediT authorship contribution statement

**Lalu Muhammad Irham:** Formal analysis, Data curation, Conceptualization, Writing – original draft, Writing – review & editing, Methodology, Visualization. **Dwi Hilda Putri:** Methodology, Writing – original draft. **Bramadi Arya:** Writing – review & editing, Software, Writing – original draft. **Wirawan Adikusuma:** Formal analysis, Writing – original draft. **Rita Maliza:** Conceptualization, Methodology, Writing – review & editing, Formal analysis, Writing – original draft, Funding acquisition.

## Ethics approval

Ethical approval was not required for this study because it exclusively involved the analysis of data obtained from publicly accessible databases. The study did not involve direct participation of human subjects, the collection of new personal data, or access to identifiable individual-level information.

## Declaration of competing interest

The authors declare that they have no known competing financial interests or personal relationships that could have appeared to influence the work reported in this paper.

## Data Availability

All data generated or analyzed during this study are included in this published article and its supplementary information files.
